# Gut bacterial communities and their assembly processing in *Cnaphalocrocis medinalis* from different geographic sources

**DOI:** 10.3389/fmicb.2022.1035644

**Published:** 2022-12-15

**Authors:** Yajun Yang, Xiaogai Liu, Jiawen Guo, Hongxing Xu, Yinghong Liu, Zhongxian Lu

**Affiliations:** ^1^State Key Laboratory for Managing Biotic and Chemical Treats to the Quality and Safety of Agro-products, Institute of Plant Protection and Microbiology, Zhejiang Academy of Agricultural Sciences, Hangzhou, China; ^2^College of Plant Protection, Southwest University, Chongqing, China

**Keywords:** rice leaffolder, gut microbiota, function, geography, selection, ecological processes

## Abstract

**Introduction:**

The insect gut harbors numerous microorganisms that may have functions in development and reproduction, digestion, immunity and protection, and detoxification. Recently, the influence factors on gut microbiota were evaluated in the rice leaffolder *Cnaphalocrocis medinalis*, a widespread insect pest in paddy fields. However, the relationship between gut microbiota composition and geography is poorly understood in *C. medinalis*.

**Methods:**

To reveal the patterns of *C. medinalis* gut bacterial communities across geographic sources and the ecological processes driving the patterns, *C. medinalis* were sampled from six geographic sources in China, Thailand, and Vietnam in 2016, followed by gut bacterial 16S ribosomal RNA gene sequencing.

**Results:**

A total of 22 bacterial phyla, 56 classes, 84 orders, 138 families, 228 genera, and 299 species were generated in *C. medinalis* from six geographic sources. All alpha diversity indices differed among the samples from different geographic sources. Analysis of similarity (ANOSIM) and permutational multivariate analysis of variance (PERMANOVA) both revealed significant differences in the gut microbiota of *C. medinalis* from six geographic sources. A total of 94 different taxa were screened as indicators for the gut microbiota of *C. medinalis* from six geographic sources by linear discriminant analysis effect size (LEfSe). The gene ontology (GO) pathways of the gut microbiota in *C. medinalis* differed among geographic sources. In total, the bacterial communities within geographic sources were mainly determined by stochastic processes, and those between geographic sources were mainly determined by deterministic processes.

**Discussion:**

This study elucidates that geography plays a crucial role in shaping the gut microbiota of *C. medinalis*. Thus, it enriches our knowledge of gut bacteria in *C. medinalis* and sheds light on the mechanisms underlying *C. medinalis* gut microbial shifts across geography.

## Introduction

The insect gut is colonized by numerous microorganisms (Douglas, 2015), and these microorganisms have been reported to contribute to many important functions, including development and reproduction, nutrient metabolism, pathogen and insecticidal resistance, immunity, digestion, and detoxification (Dillon and Dillon, 2004; Anand et al., 2010; Engel and Moran, 2013; Beran and Gershenzon, 2016; Cheng et al., 2017; Voirol et al., 2018; Blanton and Peterson, 2020; Pyszko et al., 2020; Li et al., 2021; Moore and Aparicio, 2022). In the fruit fly (*Drosophila melanogaster*), microbiome interactions were reported to shape host fitness (Gould et al., 2018). Core gut symbiotic bacteria—*Lactococcus*, *Enterococcus*, *Serratia*, and *Dysgonomonas*—isolated from the bamboo snout beetle *Cyrtotrachelus buqueti* have the capability of degrading lignocellulose (Luo et al., 2019). The gut microbiota is crucial in regulating the interconnections between both the host and gut pathogens, and the interaction of the gut microbiota and pathogenic fungi could result in the acceleration of mosquito mortality (Wei et al., 2017). However, the gut microbiota facilitates the weight gain of honeybees through bacterial metabolism and hormonal signaling, and increases the fitness of the pine weevil by degrading conifer diterpenes (Berasategui et al., 2017; Zheng et al., 2017). Gut symbionts could enhance resistance to trichlorphon, an organophosphate insecticide in the oriental fruit fly *Bactrocera dorsalis* (Hendel) (Cheng et al., 2017). Likewise, gut bacteria were proven to increase the resistance of the silkworm *Bombyx mori* against chlorpyrifos, another organophosphate insecticide (Chen et al., 2020). The honeybee (*Apis mellifera*) intestinal microbiota could promote host endogenous detoxification ability by regulating P450 gene expression in the digestive tract (Wu et al., 2020). In a *Plutella xylostella* population susceptible to Cry1Ac protoxin, the gut microbiota is correlated with the host immunological response (Li et al., 2021). Considering the importance of insect gut microbiota, application in gut symbiont-driven pest control may be a novel concept to make insect pests more susceptible to wide population management approaches by targeting microbial symbionts (Berasategui et al., 2016). Exploiting the functions of gut microorganisms could advance knowledge of gut microbiota-insect interactions and facilitate the application of gut symbiont-driven pest management (Frago et al., 2012; Berasategui et al., 2016; Kyritsis et al., 2017).

Many factors can influence the composition and diversity of gut microbiota including development stages, diet, pH, temperature and humidity, and geographic source (Schmitt-Wagner et al., 2003; Behar et al., 2008; Mikaelyan et al., 2015; Jones et al., 2018; Yang et al., 2020; Jaramillo and Castañeda, 2021). Bosmans et al. (2018) found that land-use change could cause differences in the gut microbial communities in bumblebees (*Bombus terrestris*). Jones et al. (2019) suggested that the host plant is the main motivation to shape intestinal microbiota, while variations in insect physiology, intestinal tract, and local external conditions could be responsible for differences in microbiota. The relative abundances of anaerobes were significantly different in insects, and the gut bacterial diversity was impacted by host environmental location, food, growth status, and phylogeny (Yun et al., 2014). Liu et al. (2021) reported that larval bacterial communities differed between laboratory and wild populations of ghost moths (*Thitarodes* sp.). Yu et al. (2021) compared the microbiota in *Leptinotarsa decemlineata* (Say), Colorado potato beetles, derived from distinct geographic locations in China, and found that order and genus were proper taxonomic categories to discriminate the geographical locations of Colorado potato beetles. Honey bee colonies from different habitats could harbor significantly different gut microbiota (Jones et al., 2018). Similarly, the composition and diversity of gut microbiota differed in large black chafer (*Holotrichia parallela*) populations from various geographical sources (Huang and Zhang, 2013).

The rice leaffolder *Cnaphalocrocis medinalis* (Guenée; Lepidoptera: Crambidae), which mainly feeds on rice plants (*Oryza sativa* L.), is a significant insect pest in Asia and might result in tremendous financial losses to rice production (Barrion et al., 1991; Cheng, 1996; Yang et al., 2015). This insect is an important long-distance migratory insect pest and cannot overwinter in temperate climates (Cheng, 1996). The *C. medinalis* population migrates northward beginning in March every year from the southern areas, expanding their regional distributions to cover the rice production zones in China, the Korean Peninsula, and Japan, and their direction becomes predominantly southbound from September onward (Chang et al., 1980; Riley et al., 1995; Wang et al., 2017). In 2015, *C. medinalis* devastated 15.5 million hectares of rice plants, leading to a yield reduction of 0.47 million tons in China (Yang et al., 2015). A growing number of environmentally friendly alternatives and suggestions for controlling *C. medinalis* have been researched and put forth, despite the fact that the main method of management for this insect pest relied on chemicals (Yang et al., 2015; Kalaivani et al., 2018; Senthil-Nathan, 2019; Ramasubramanian et al., 2022). Several microorganisms including *Bacillus thuringiensis*, *Bacillus subtilis*, *Metarhizium anisopliae*, and *Beauveria bassiana* were evaluated their capacity to suppress *C. medinalis* (Kandibane et al., 2010; Kirubakaran et al., 2014; Shakir et al., 2015; Ramasubramanian et al., 2022). Given the importance of microbes, investigation on the gut bacteria of *C. medinalis* will contribute to the understanding of insect ecology, and assist the development of innovative pest management approaches based on the application of microorganisms. The gut microbiota in *C. medinalis* larvae was characterized by traditional isolation and culture methods and Illumina MiSeq technology (Yang, 2012; Liu et al., 2016). The structure and composition of the gut microbiota in *C. medinalis* were analyzed across developmental stages (Yang et al., 2020). In this study, to assess the patterns of *C. medinalis* gut microbial communities across geographic sources and the ecological processes generating the patterns, *C. medinalis* were sampled from six geographic sources in China, Thailand, and Vietnam, followed by bacterial 16S ribosomal RNA gene sequencing with the Illumina MiSeq technology. The results of this study will add to our knowledge of the gut microbiota of *C. medinalis*, and provide fresh insights into *C. medinalis* population features.

## Materials and methods

### Sample preparation

*Cnaphalocrocis medinalis* adults were collected from paddy fields from four sites in China, one site in Thailand, and one site in Vietnam in 2016. Detailed information on the collected populations is listed in Table 1. This study was conducted based on the populations collected in 2016, and the fifth-instar larvae derived from the eggs laid by the collected moths were used in the study. The collected adults were cultivated with 10% honey solution in the laboratory under controlled circumstances of 26 ± 1°C temperature, 70 ± 10% relative humidity, and a photoperiod of 16:8 (L:D) h. The larvae derived from each geographic source were fed rice leaves. Rice plants were potted in the greenhouse. Rice plant leaves were washed with sterile ddH_2_O, then air dried before being fed to *C. medinalis* larvae, and adequate rice leaves were prepared for the *C. medinalis* larvae.

**Table 1:** Collection and sequencing of *Cnaphalocrocis medinalis* samples.

Country	Specific sources	Sample code	Latitude	Longitude	Collection year
Thailand	Chainat	TH	14	101	2016
Vietnam	Ho Chi Minh City	VN	11	107	2016
China	Nanning	NN	23	108	2016
China	Leshan	LS	29	104	2016
China	Changsha	CS	28	113	2016
China	Hangzhou	HZ	30	120	2016

One-day fifth instar larvae of *C. medinalis* with similar size were chosen at random to dissect intestinal tract from each geographic source for investigation. A biological sample was created by pooling 20 guts, and three duplicates were established for each geographic source. The larvae of *C. medinalis* were washed with sterile ddH_2_O before being disinfected with 75% ethanol for 90s, and then rinsed again with sterile ddH_2_O. The intestines were collected after dissection and stored at −80°C in 1.5 ml sterile tubes until use.

### DNA extraction, PCR, and sequencing

The dissected intestines of *C. medinalis* were homogenized with 0.5 ml of phosphate buffer saline (PBS buffer, pH 7.5) by shaking with sterile glass beads (0.5 mm diameter) in a sterile tube for 15 min on a vortex. Total DNA were isolated from the samples using the bacterial DNA extraction kit (E.Z.N.A.^®^, OMEGA, USA) according to the manufacturer’s instructions. Amplicons were generated in a 20 μl reaction system with 5 × FastPfu Buffer (4 μl), 2.5 mM dNTPs (2 μl), each primer (5 μM, 0.8 μL), FastPfu Polymerase (0.4 μl), and sample DNA(10 ng) by PCR (95°C for 2 min, followed by 25 cycles at 95°C for 30 s, 55°C for 30 s, and 72°C for 30 s and a final extension at 72°C for 5 min) of V4-V5 regions of the bacterial 16S ribosomal RNA gene using the primers 515F (5′-GTGCCAGCMGCCGCGG-3′) and 907R (5′-CCGTCAATTCMTTTRAGTTT-3′). Amplicons were extracted and purified using the AxyPrep DNA Gel Extraction Kit (Axygen Biosciences, Union City, CA, U.S.). Then, they were measured using QuantiFluor™-ST (Promega, U.S.), pooled in equimolar amounts, and paired-end sequenced (2 × 250) using standard methods on an Illumina MiSeq platform.

### Data analyses

Raw sequencing data with FASTQ format were demultiplexed and quality-filtered using QIIME (version 1.17).^1^ Based on the sequence similarity, effective sequences were classified into multiple operational taxonomic units (OTUs) at a similarity level of 97% with UPARSE (version 7.1),^2^ and then chimeric sequences were identified and removed with UCHIME.^3^ All of the sequences were annotated and blasted against the Silva (SSU115) 16S ribosomal RNA database with a confidence level of 70% for each 16S ribosomal RNA gene sequence assessed through RDP Classifier.^4^

To assess alpha diversity, five parameters were used, including OTU number, ACE, Chao1, Shannon, and Simpson’s index. The alpha diversity indices and relative abundance data were subjected to one-way analysis of variance (ANOVA) with SPSS 26.0 (IBM SPSS Statistics), and multiple comparisons were assessed with Tukey’s test. Rarefaction curves were graphed with R software.^5^ Principal coordinate analysis (PCoA) was performed among all the samples from different geographic sources based on pairwise weighted UniFrac distances and Bray–Curtis distances. Nonmetric multidimensional scaling (NMDS) plots were drawn based on Bray–Curtis distance metrics. Additionally, an analysis of similarity (ANOSIM) was performed to assess the differences in the composition of microbiota across samples from six geographic sources. Individual repetitions were included in the model as a random effect, and 999 permutations were used to construct the permutational multivariate analysis of variance (PERMANOVA). R software^6^ was used to analyze or plot the data for PCoA, NMDS, ANOSIM, and PERMANOVA. Linear discriminant analysis (LDA) was applied to assess the biomarkers for significant variations among samples from six geographic sources with LDA scores larger than two. Galaxy^7^ was used to generate a cladogram to reveal the distribution of the significant biomarkers at various taxonomic levels. Functional predictions were performed for the gut microbiota through annotating gene ontology (GO) pathways of OTUs against the Ref99NR database employing the Tax4Fun2 package in R software.

Ecological processing of the microbial communities and phylogenetic turnover within the system was conducted following the framework of Stegen et al. (2013). In this framework, two distinct metrics were utilized to evaluate the contributions of selection, dispersal, and drift that drive an observed assembly of the bacterial community. First, the strength of selection was evaluated by assessing variations in phylogenetic distance based on beta-mean nearest taxon distance (βMNTD) and the β-nearest taxon index (βNTI) for all pairwise comparisons within the samples. Following the confirmation of the roles of selective processes, pairwise comparisons without significant βNTI metrics were evaluated through the Raup-Crick bray (RCbray) index metric to reflect the strength of dispersal on assembly. βMNTD and βNTI were performed using R software with the codes provided by Stegen et al. (2013).^8^ RCbray index was performed through R software with the codes provided by Stegen et al. (2013).^9^ Supplementary Table S1 provides a thorough explanation of ecological processing (Stegen et al., 2015).

## Results

### Sequence analysis and taxa generation

We sequenced the gut microbiota of *C. medinalis* larvae derived from six geographic sources (Table 1) and retrieved a total of 1,000,338 trimmed paired reads (Supplementary Table S2). Among these reads, 558 OTUs were identified. The high values of Good’s coverage index in all the samples from geographic sources reflected that the sequencing depth was adequate to profile the microbial communities (Supplementary Table S2). Rarefaction curves based on 16S ribosomal RNA gene sequences indicated that the majority of the OTUs were detected (Supplementary Figure S1). A total of 22 bacterial phyla, 56 classes, 84 orders, 138 families, 228 genera, and 299 species were generated in *C. medinalis* from six geographic sources (Table 2).

**Table 2:** Number of gut bacterial taxonomic categories in *Cnaphalocrocis medinalis* from different geographic sources.

Source	phylum	class	order	family	genus	species
LS	21	51	78	123	191	248
NN	16	38	59	92	141	169
CS	14	33	50	79	115	138
HZ	12	31	50	83	125	141
TH	5	8	17	35	57	64
VN	11	27	44	72	114	134
Total	22	56	84	138	228	299

### Composition of the gut microbiota in *Cnaphalocrocis medinalis* from different geographic sources

**Figure 1:**
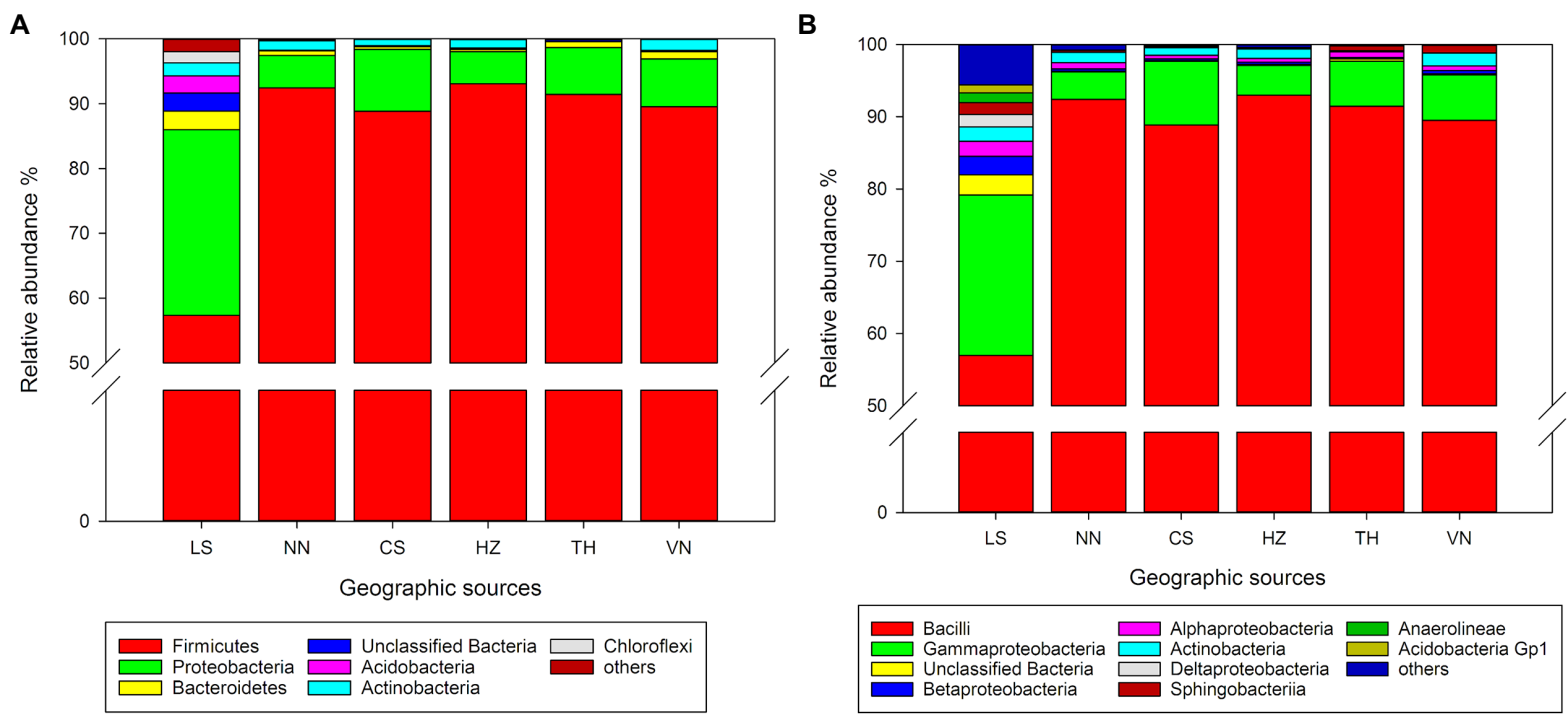
Relative abundance of the gut microbiota in *C. medinalis* from different geographic sources at the phylum **(A)** and class **(B)** levels.

At the phylum level, *Firmicutes* and *Proteobacteria* are the dominant taxa in the gut microbiota of *C. medinalis* from different geographic sources (Figure 1A). The relative abundance of *Firmicutes* varied from 57.31 to 93.08%, and the highest relative abundance was observed in the HZ population, followed by the NN, TH, VN, CS, and LS populations. The relative abundance of *Proteobacteria* varied from 4.94 to 28.66%, and the highest relative abundance was observed in the LS population, followed by the NN, TH, VN, CS, and HZ populations.

At the class level, *Bacilli* and *Gammaproteobacteria* were the dominant taxa in the gut microbiota of *C. medinalis* from six geographic sources (Figure 1B). *Actinobacteria*, *Alphaproteobacteria*, *Bacilli*, *Betaproteobacteria*, *Gammaproteobacteria*, *Sphingobacteriia*, and unclassified *Bacteria* occupied the top 10 taxa in the samples from all six geographic sources. The relative abundance of *Bacilli* varied from 56.99 to 92.98%, and the highest relative abundance was observed in the HZ population, followed by the NN, TH, VN, CS, and LS populations. The relative abundance of *Gammaproteobacteria* varied from 3.78 to 22.17%, and the highest relative abundance was observed in the LS population, followed by the CS, VN, TH, HZ, and NN populations. The relative abundance of *Betaproteobacteria* varied from 0.21 to 2.55%, and the highest relative abundance was observed in the LS population, followed by the VN, NN, HZ, CS, and TH populations.

**Figure 2:**
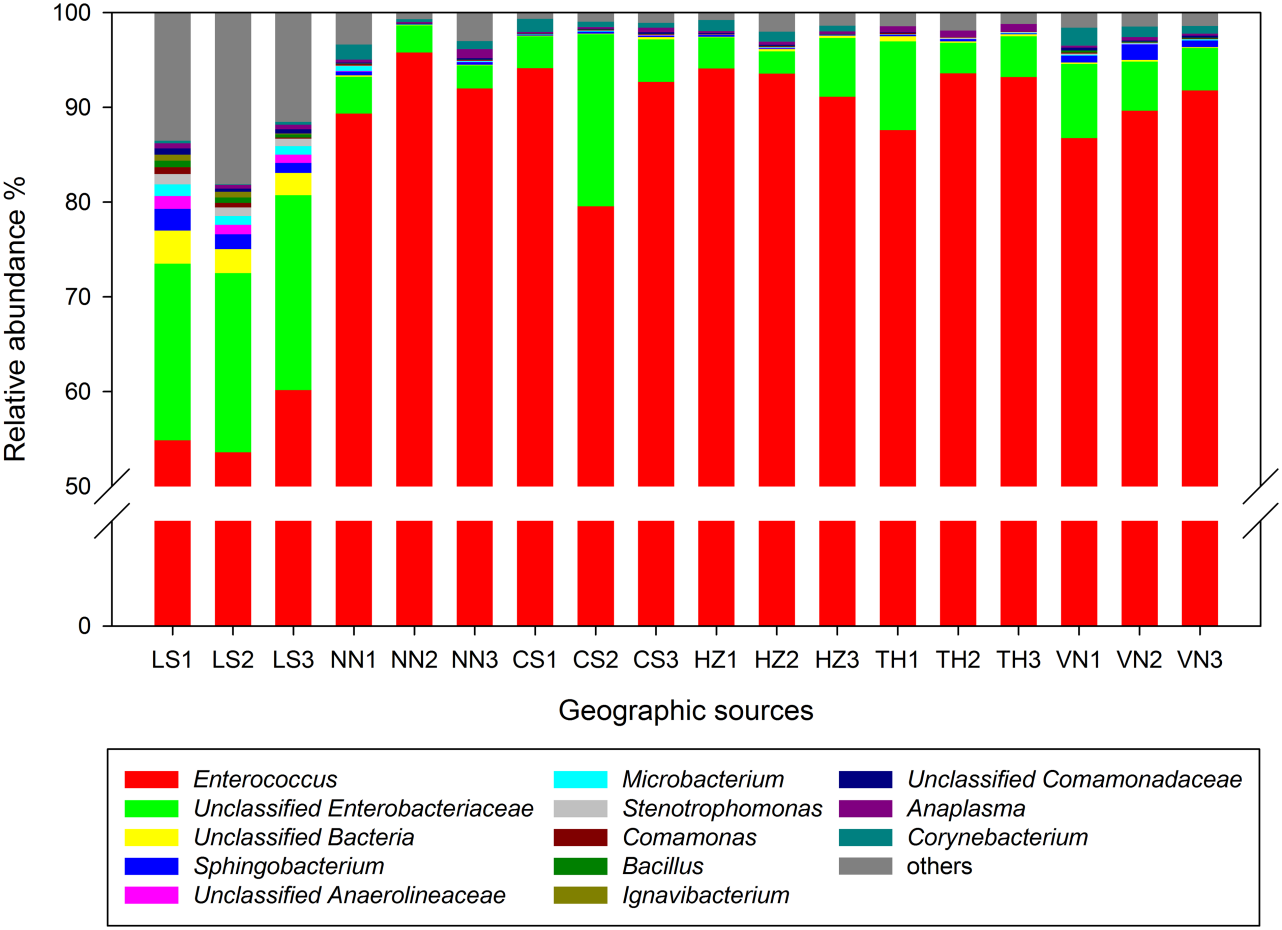
Relative abundance of the gut microbiota in *C. medinalis* from different geographic sources at the genus level.

At the genus level, *Enterococcus* and unclassified *Enterobacteriaceae* were the dominant taxa in the gut microbiota of *C. medinalis* from six geographic sources (Figure 2). The relative abundance of *Enterococcus* varied from 56.20 to 92.92%, and the highest relative abundance was observed in the HZ population, followed by the NN, TH, VN, CS, and LS populations. The relative abundance of unclassified *Enterobacteriaceae* varied from 3.06 to 19.36%, and the highest relative abundance was observed in the LS population, followed by the CS, VN, TH, HZ, and NN populations.

### Alpha diversity of the gut microbiota in *Cnaphalocrocis medinalis* from different geographic sources

Alpha diversity indices were analyzed to clarify the differences in the richness and diversity of the bacterial communities of *C. medinalis* from different geographic sources. The OTU numbers of *C. medinalis* samples from six geographic sources ranged from 54 to 311, the Ace index ranged from 58.50 to 322.67, the Chao1 index ranged from 61.59 to 319.94, the Shannon index ranged from 0.36 to 3.59, and the Simpson index ranged from 0.32 to 0.92 (Supplementary Table S3). The OTU number and the Shannon index were considerably greater in the LS population than in the other populations, Ace was higher in the LS population than in the HZ and TH populations, the Chao1 index was higher in the LS population than in the CS, HZ, TH, and VN populations, and the Simpson index was lower in the LS population than in the other populations (Table 3). Analysis of variance (ANOVA) revealed that all alpha diversity indices exhibited significant differences across samples from distinct geographic sources (OTU numbers: *F* = 14.969, *p* = 8.45E-05; Ace: *F* = 6.050, *p* = 0.005; Chao1: *F* = 5.396, *p* = 0.008; Shannon: *F* = 54.464, *p* = 7.84E-08; Simpson: *F* = 27.618, *p* = 3.44E-06; Supplementary Table S4).

**Table 3:** Alpha diversity indices of gut bacterial communities in *Cnaphalocrocis medinalis* from different geographic sources.

Geographic sources	Alpha diversity indices^1^				
	OTU number	ACE	Chao1	Shannon	Simpson
LS	273.3 ± 19.5a	289.16 ± 15.70a	299.43 ± 11.62a	3.28 ± 0.23a	0.35 ± 0.02b
NN	133.3 ± 28.7b	204.06 ± 52.62ab	206.30 ± 57.93ab	0.69 ± 0.17b	0.85 ± 0.03a
CS	92.3 ± 19.1b	136.43 ± 44.68ab	135.07 ± 41.25b	0.69 ± 0.16b	0.80 ± 0.07a
HZ	91.3 ± 15.8b	126.74 ± 17.50b	125.24 ± 21.41b	0.60 ± 0.04b	0.87 ± 0.02a
TH	55.0 ± 1.0b	62.39 ± 0.26b	59.73 ± 0.62b	0.60 ± 0.08b	0.84 ± 0.03a
VN	108.0 ± 23.4b	147.93 ± 36.65ab	137.15 ± 33.03b	0.81 ± 0.07b	0.80 ± 0.02a

^1^Data are shown as the mean ± SE. The same lowercase letter following the data in the same column indicates that there were no significant differences between the groups at a level of *p* < 0.05 by Tukey’s test.

### Beta diversity of the gut microbiota in *Cnaphalocrocis medinalis* from different geographic sources

**Figure 3:**
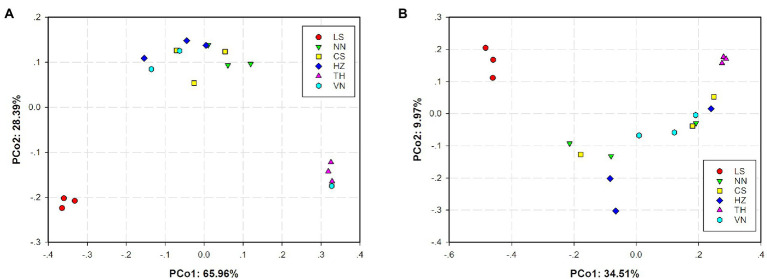
Principal coordinate analysis (PCoA) of bacterial communities in *C. medinalis* from different geographic sources based on weighted UniFrac **(A)** and Bray–Curtis **(B)** distances.

**Figure 4:**
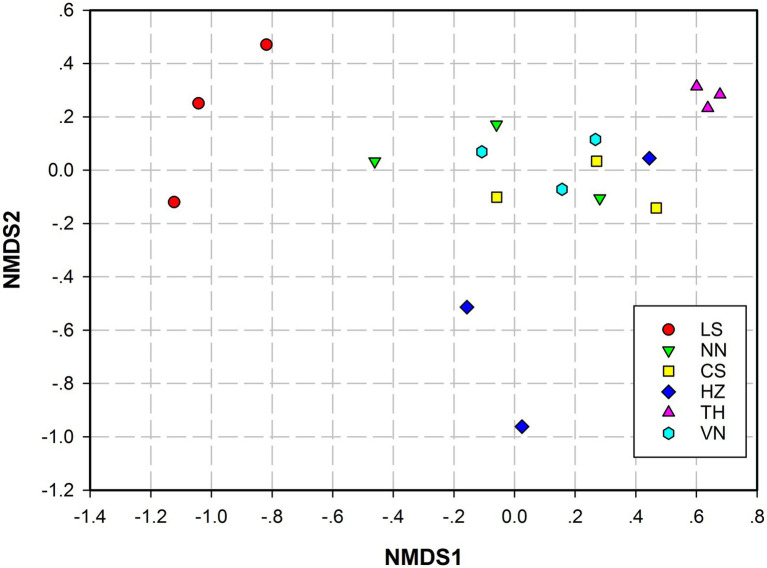
Nonmetric multidimensional scaling (NMDS) analysis of the bacterial communities in different *C. medinalis* geographic populations. NMDS plots were constructed using Bray–Curtis. Stress values (stress = 0.079) indicate a good fit.

Principal coordinates analysis (PCoA) based on the weighted UniFrac distance and Bray–Curtis distance was conducted to present the bacterial community similarities between samples from different geographic sources. The abscissa and ordinate of the PCoA scatter plot represented the two characteristic values contributing the most to variations between the samples from six geographic sources, with effect degrees of 34.51 and 9.97% based on weighted UniFrac distance (Figure 3A) and 65.96 and 28.39% based on Bray–Curtis distance (Figure 3B), respectively. Significant differences in the gut microbiota of *C. medinalis* from different geographic sources were observed based on permutational multivariate analysis of variance (PERMANOVA; *R*^2^ = 0.25, *p* = 0.009). Nonmetric multidimensional scaling (NMDS) analysis demonstrated that the gut microbiota of *C. medinalis* from different geographic sources differed significantly (Figure 4). Analysis of similarity (ANOSIM) revealed significant differences in the gut microbiota of *C. medinalis* from different geographic sources (*R* = 0.544, *p* = 0.002).

### Linear discriminant analysis effect size on the gut microbiota in *Cnaphalocrocis medinalis* from different geographic sources

**Figure 5:**
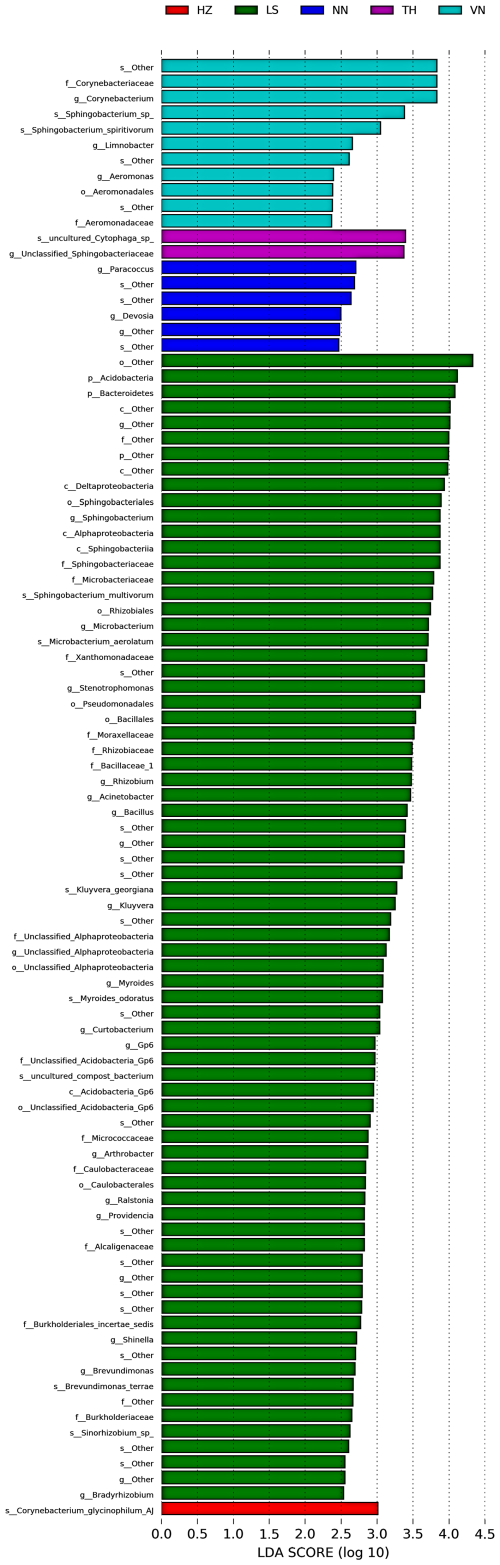
Bacterial taxa with linear discriminant analysis (LDA) scores greater than two in the gut microbiota of *C. medinalis* from different geographic sources.

**Figure 6:**
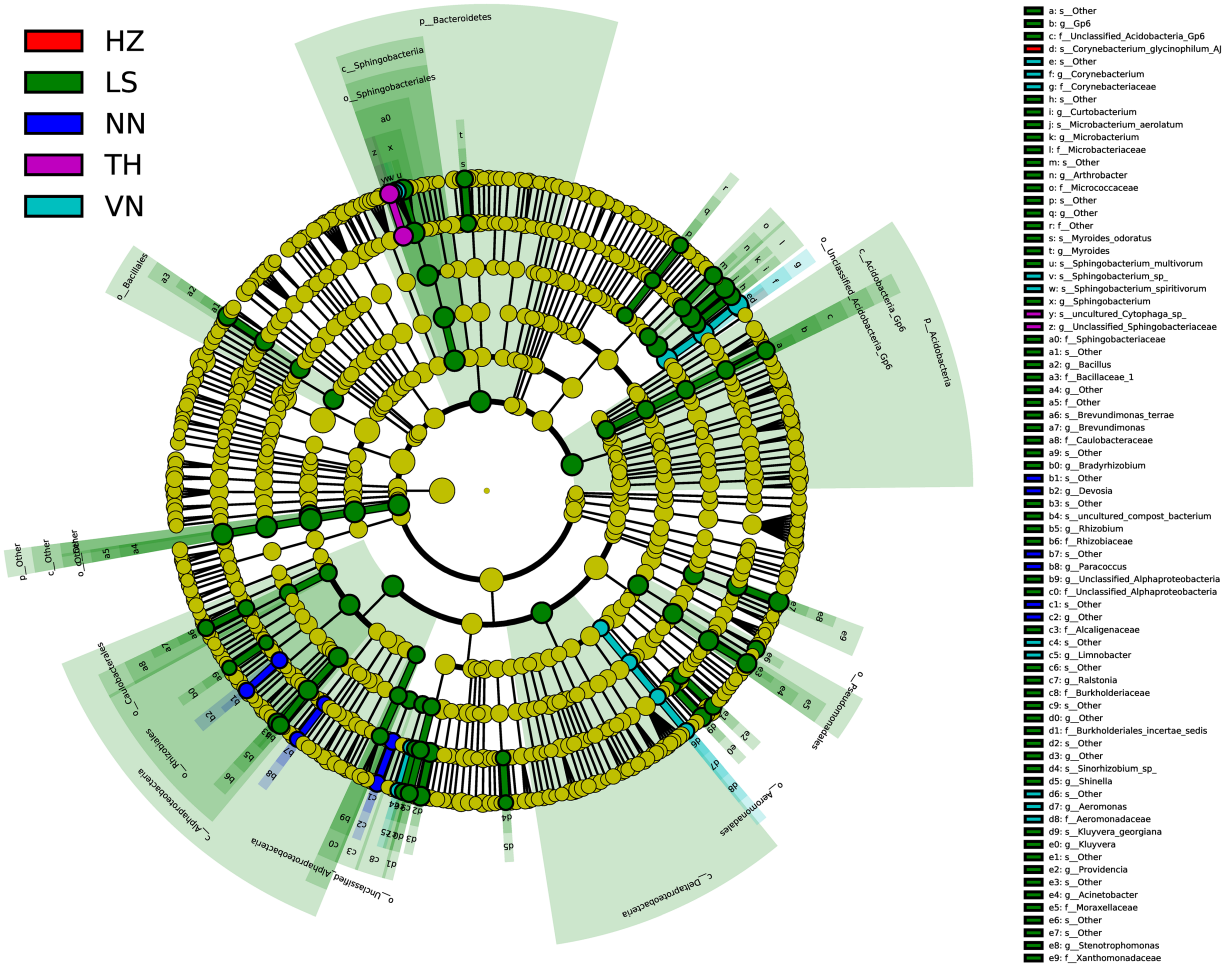
Cladogram of bacterial biomarkers, from the phylum (innermost ring) to species (outermost ring) level, with an LDA score > 2. Differential bacterial taxa are marked by lowercase letters. Each small circle at different taxonomic levels represents a taxon at that level, and the diameter of the circle is proportional to the relative abundance. The coloring principle is to color the taxa with no significant difference as yellow and the other different taxa as the group with the highest abundance of the species. Different colors represent different groups, and nodes with different colors represent the communities that play an important role in the group represented by the color.

To discover significant biomarkers among samples from different geographic sources, linear discriminant analysis (LDA) effect size (LEfSe) was applied to screen out distinct taxa from kingdom to species levels among samples from six geographic sources based on a standard of LDA > 2 (Figure 5). Simultaneously, a cladogram was drawn to overall understand the distribution of these various taxa at different levels from phylum to species (Figure 6). There were 94 different taxa mainly belonging to *Proteobacteria, Firmicutes*, *Bacteroidetes*, *Actinobacteria*, and other *Bacteria* in the gut microbiota of *C. medinalis* from different geographic sources (LDA > 2). Most of the different taxa (LDA > 2) were found in the gut microbiota of the LS population. Six taxa belonging to *Proteobacteria*, two taxa belonging to *Bacteroidetes*, and three taxa belonging to *Actinobacteria* were found in the gut microbiota of the VN population (LDA > 2). Two taxa belonging to *Bacteroidetes* were found in the gut microbiota of the TH population (LDA > 2). Six taxa belonging to *Proteobacteria* were found in the gut microbiota of the NN population (LDA > 2). A total of 39 taxa belonging to *Proteobacteria*, eight taxa belonging to *Bacteroidetes*, 17 taxa belonging to *Actinobacteria*, four taxa belonging to *Firmicutes*, and six taxa belonging to *other Bacteria* were found in the gut microbiota of the LS population (LDA > 2). One taxon belonging to *Actinobacteria* was found in the gut microbiota of the HZ population (LDA > 2). There were no different taxa found in the gut microbiota of the CS population (LDA > 2).

There were 52 different taxa among samples from different geographic sources on a standard of LDA > 3, including two taxa belonging to *Bacteroidetes* and three taxa belonging to *Actinobacteria* in the gut microbiota of the VN population, two taxa belonging to *Bacteroidetes* in the gut microbiota of the TH population, 20 taxa belonging to *Proteobacteria*, eight taxa belonging to *Bacteroidetes*, five taxa belonging to *Actinobacteria*, four taxa belonging to *Firmicutes*, one taxon belonging to *Acidobacteria*, and six taxa belonging to *other Bacteria* in the gut microbiota of the LS population, and one taxon belonging to *Actinobacteria* in the gut microbiota of the HZ population.

Five different taxa were found in the gut microbiota among samples from different geographic sources on a standard of LDA > 4, including one taxon belonging to *Actinobacteria*, one taxon belonging to *Bacteroidetes*, and three taxa belonging to *other Bacteria* in the gut microbiota of the LS population.

### Functional prediction of the gut microbiota in *Cnaphalocrocis medinalis* from different geographic sources

**Figure 7:**
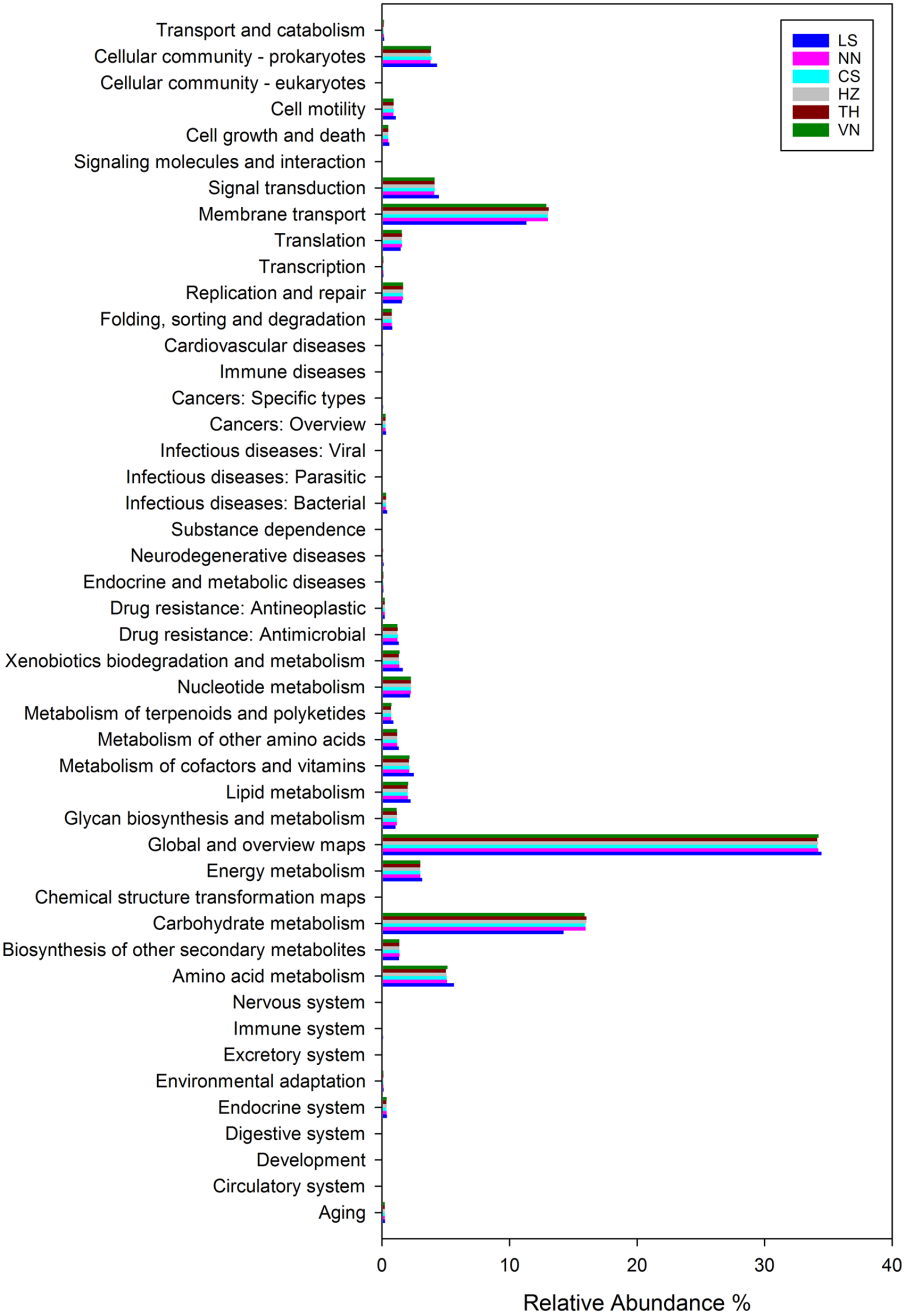
Comparison of predicted GO functions of the gut bacteria in *C. medinalis* from different geographic sources.

To reveal the significant role of the gut microbiota of *C. medinalis* from six geographic sources, the function of the gut microbiota in *C. medinalis* from different geographic sources was predicted using R software with Tax4Fun2 based on 16S ribosomal RNA gene sequencing data and the Ref99NR database (Figure 7). Functional prediction demonstrated that metabolism (70.22–70.53%) was the most functional category, followed by environmental information processing (15.79–17.21%), cellular process (5.29–6.12%), genetic information processing (3.90–4.06%), human diseases (2.32–2.72%), and organismal systems (0.82–0.94%).

The highest abundance was found in gut microbiota functioning in global and overview maps (34.12–34.45%) among the categories of metabolism, followed by those functioning in carbohydrate metabolism (14.23–16.04%), amino acid metabolism (4.99–5.62%), energy metabolism (2.98–3.13%), nucleotide metabolism (2.18–2.26%), metabolism of cofactors and vitamins (2.11–2.49%), lipid metabolism (2.00–2.24%), xenobiotics biodegradation and metabolism (1.31–1.62%), other secondary metabolites biosynthesis (1.33–1.36%), other amino acids metabolism (1.17–1.30%), glycan biosynthesis and metabolism (1.06–1.15%), terpenoids and polyketides metabolism(0.70–0.88%), and chemical structure transformation maps (0.01–0.02%). The highest abundance was found in gut microbiota functioning in membrane transport (11.33–13.07%) among the categories of environmental information processing, followed by those functioning in signal transduction (4.10–4.45%) and signaling molecules and interactions (0.01–0.02%). The highest abundance was found in gut microbiota functioning in the cellular community (3.79–4.31%) among the categories of cellular processes, followed by those functioning in cell motility (0.88–1.07%), cell growth and death (0.48–0.57%), and transport and catabolism (0.14–0.17%). The highest abundance was found in gut microbiota functioning in replication and repair (1.55–1.65%) among the categories of genetic information processing, followed by those functioning in translation (1.46–1.55%), folding, sorting, and degradation (0.75–0.80%), and transcription (0.09–0.10%). The highest abundance was found in gut microbiota functioning in drug resistance: antimicrobial (1.19–1.29%) among the categories of human diseases, followed by those functioning in infectious diseases: bacterial (0.30–0.40%), cancers: overview (0.26–0.31%), drug resistance: antineoplastic (0.21%), endocrine and metabolic diseases (0.10–0.11%), neurodegenerative diseases (0.07–0.11%), cardiovascular diseases (0.06–0.09%), cancers: specific types (0.05–0.08%), infectious diseases: viral (0.04–0.05%), and immune diseases (0.03%). The highest abundance was found in gut microbiota functioning in the endocrine system (0.35–0.37%) among the categories of organismal systems, followed by those functioning in aging (0.19–0.22%), environmental adaptation (0.10–0.12%), the immune system (0.07–0.08%), the nervous system (0.06–0.07%), and the digestive system (0.05%).

At level 1, the top 20 GO functional pathways of the gut microbiota of *C. medinalis* from six geographic sources had relative abundances of 63.96–67.91% (Table 4). The relative abundances of the gut microbiota functioning in microbial metabolism in diverse environments, two-component systems, and quorum sensing were all considerably greater in the LS population than in the other geographic populations. The relative abundances of the gut microbiota functioning in ABC transporters, the phosphotransferase system (PTS), starch and sucrose metabolism, amino acids biosynthesis, amino sugar and nucleotide sugar metabolism, glycolysis/gluconeogenesis, fructose and mannose metabolism, purine metabolism, pyruvate metabolism, ribosome, pyrimidine metabolism, and cysteine and methionine metabolism in the LS population of *C. medinalis* were significantly lower than those in other geographic populations. PERMANOVA demonstrated significant variations in the GO pathways of the gut microbiota of *C. medinalis* from different geographic sources (*R*^2^ = 0.25, *p* = 0.009). ANOSIM results revealed substantial changes in the GO pathways of the gut microbiota of *C. medinalis* from different geographic sources (*R* = 0.3169, *p* = 0.007).

**Table 4:** The relative abundance of the top 20 GO pathway functions (Level 1) of the gut microbiota of *Cnaphalocrocis medinalis* from geographic sources.

Level 1	The relative abundance (%)^1^					
	LS	NN	CS	HZ	TH	VN
Metabolic pathways	13.13 ± 0.003 a	13.10 ± 0.02ab	13.09 ± 0.002b	13.09 ± 0.004ab	13.09 ± 0.004ab	13.10 ± 0.01ab
ABC transporters	6.88 ± 0.06b	7.43 ± 0.04a	7.41 ± 0.03a	7.41 ± 0.02a	7.41 ± 0.01a	7.38 ± 0.02a
Biosynthesis of secondary metabolites	5.53 ± 0.003a	5.52 ± 0.01a	5.50 ± 0.004a	5.51 ± 0.004a	5.51 ± 0.004a	5.52 ± 0.01a
Phosphotransferase system (PTS)	4.01 ± 0.08b	5.22 ± 0.11a	5.21 ± 0.11a	5.27 ± 0.03a	5.29 ± 0.04a	5.15 ± 0.04a
Microbial metabolism in diverse environments	4.94 ± 0.01a	4.80 ± 0.02b	4.81 ± 0.02b	4.81 ± 0.01b	4.79 ± 0.01b	4.82 ± 0.01b
Biosynthesis of antibiotics	4.44 ± 0.01a	4.40 ± 0.01ab	4.37 ± 0.01b	4.39 ± 0.01b	4.37 ± 0.01b	4.40 ± 0.004ab
Two-component system	4.07 ± 0.05a	3.73 ± 0.01b	3.77 ± 0.02b	3.75 ± 0.02b	3.76 ± 0.01b	3.74 ± 0.0043b
Starch and sucrose metabolism	2.39 ± 0.04b	3.08 ± 0.06a	3.06 ± 0.07a	3.11 ± 0.02a	3.11 ± 0.03a	3.04 ± 0.03a
Biosynthesis of amino acids	2.49 ± 0.01b	2.64 ± 0.01a	2.62 ± 0.02a	2.64 ± 0.01a	2.63 ± 0.01a	2.64 ± 0.01a
Quorum sensing	2.63 ± 0.01a	2.45 ± 0.02b	2.46 ± 0.03b	2.42 ± 0.004b	2.43 ± 0.01b	2.45 ± 0.01b
Amino sugar and nucleotide sugar metabolism	2.13 ± 0.03b	2.51 ± 0.04a	2.52 ± 0.03a	2.53 ± 0.01a	2.54 ± 0.01a	2.49 ± 0.02a
Carbon metabolism	2.37 ± 0.003a	2.37 ± 0.004a	2.36 ± 0.01a	2.37 ± 0.002a	2.36 ± 0.003a	2.37 ± 4E-04a
Glycolysis/Gluconeogenesis	1.85 ± 0.02b	2.12 ± 0.02a	2.13 ± 0.02a	2.14 ± 0.01a	2.14 ± 0.01a	2.11 ± 0.01a
Fructose and mannose metabolism	1.55 ± 0.03b	1.88 ± 0.03a	1.88 ± 0.03a	1.89 ± 0.01a	1.90 ± 0.01a	1.86 ± 0.01a
Purine metabolism	1.27 ± 0.003b	1.31 ± 0.01a	1.30 ± 0.01a	1.31 ± 0.002a	1.31 ± 0.003a	1.30 ± 0.002a
Pyruvate metabolism	1.09 ± 0.001b	1.12 ± 6E-04a	1.12 ± 0.01a	1.13 ± 0.002a	1.12 ± 0.003a	1.12 ± 0.001a
Ribosome	0.93 ± 0.004b	1.00 ± 0.01a	0.99 ± 0.01a	1.00 ± 0.003a	1.01 ± 0.01a	0.99 ± 0.004a
Pyrimidine metabolism	0.91 ± 0.004b	0.95 ± 0.01a	0.95 ± 0.01a	0.95 ± 0.002a	0.95 ± 0.003a	0.94 ± 0.002a
Galactose metabolism	0.79 ± 0.01b	0.97 ± 0.01a	0.96 ± 0.02a	0.98 ± 0.01a	0.98 ± 0.01a	0.96 ± 0.01a
Cysteine and methionine metabolism	0.79 ± 0.003b	0.84 ± 0.004a	0.84 ± 0.01a	0.84 ± 0.003a	0.84 ± 0.004a	0.84 ± 0.003a

^1^Data are shown as the mean ± SE. The same lowercase letter following the data in the same row indicates that there were no significant differences between the groups at a level of *p* < 0.05 by Tukey’s test.

### Ecological processing

**Figure 8:**
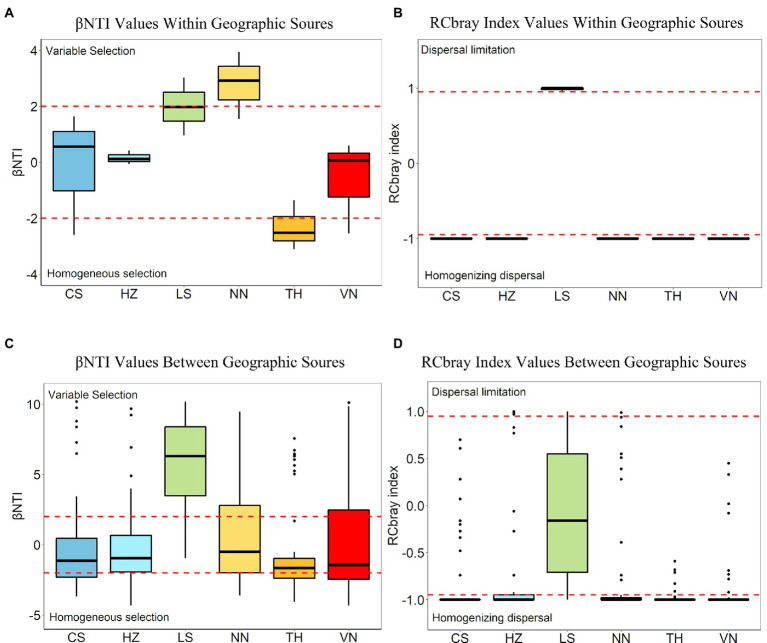
Boxplots showing the distribution of βNTI **(A)** and RCbray **(B)** values within geographic sources and the distribution of βNTI **(C)** and RCbray **(D)** values between geographic sources. Boxplots represent pairwise comparisons within geographic sources **(A,B)** and pairwise comparisons between geographic sources **(C,D)**. The dashed black line in the plots represents the null expectation. Dashed red lines in each plot represent significance thresholds for both the βNTI and RCbray metrics. Plotted RCbray values **(B,D)** do not include values from pairwise comparisons that have significant βNTI values. Boxplots display the distribution of the data, whereas the main colored box displays the interquartile range (25th to 75th percentile) of the data. Solid lines within the boxes represent the median of the data. Whiskers on the plot display the maximum and minimum expected values of the data distribution, and black-colored points represent outlier data points with respect to the plotted distribution.

**Figure 9:**
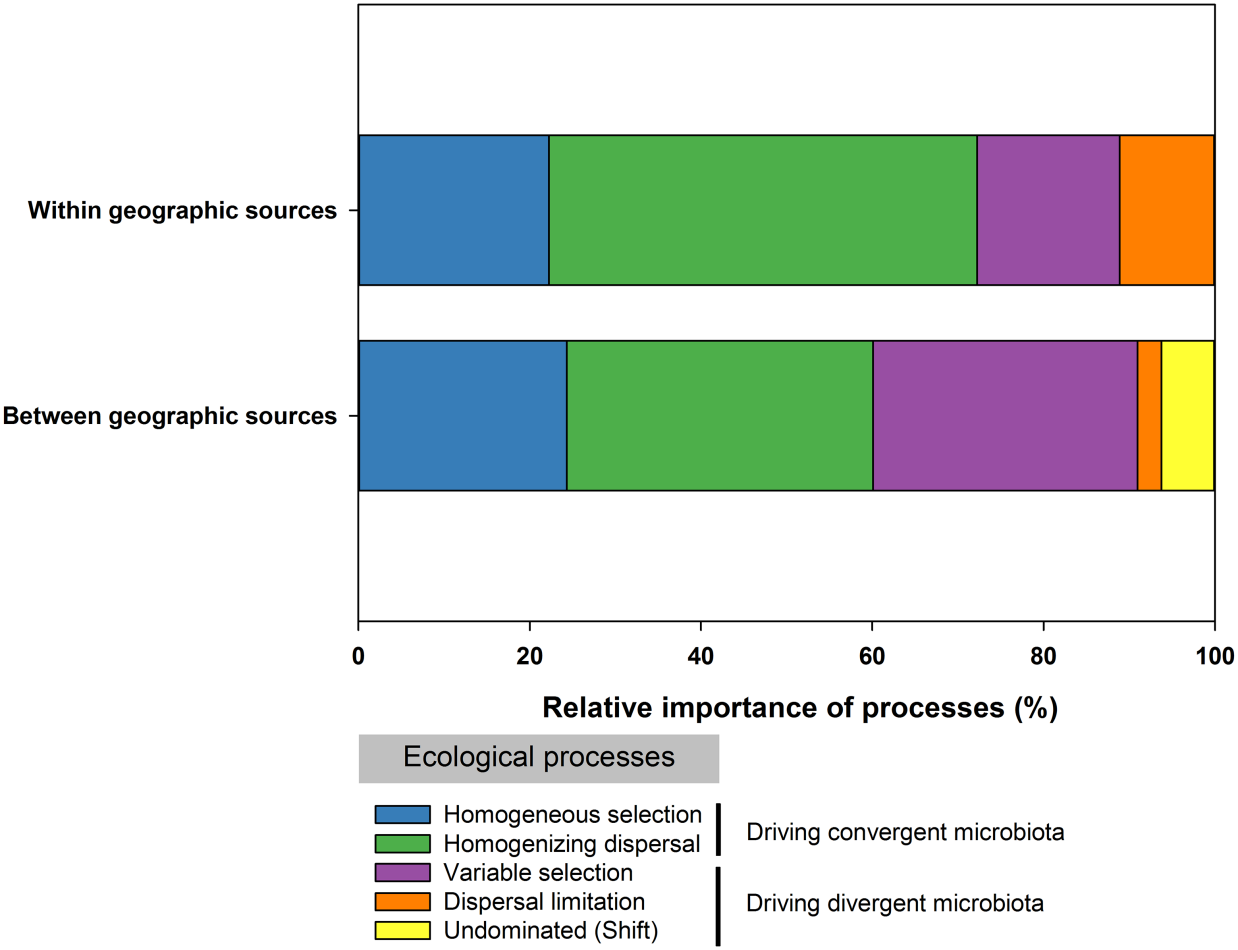
The relative importance of ecological processes in governing the gut bacterial communities of *C. medinalis* within or between geographical sources.

The assembly processes of the microbial communities were evaluated within geographic sources (Figures 8A,B) and between geographic sources (Figures 8C,D). Within geographic sources, dispersal processes and selection processes accounted for 61.11 and 38.89% of the microbial community turnover observed between samples within geographic sources, respectively (Figure 9). Variable selection plays an important role within the NN population, and homogeneous selection plays an important role within the TH population (Figures 8A,B). In pairwise comparisons within geographic sources, βNTI mean and median values close to 0 suggested that dispersal (stochastic) processes were the dominant ecological processes within the other geographic sources (Figure 8A). Overall, between geographic sources, selection processes, comprising variable selection (30.90%) and homogeneous selection (24.31%), were the dominant processes, and dispersal processes, including dispersal limitation (2.78%) and homogenizing dispersal (35.76%), accounted for 38.54% of the total (Figure 9). Variable selection strongly influenced pairwise comparisons between the LS population (77.08%) and other geographic sources. Homogenizing dispersal was the dominant ecological process affecting pairwise comparisons between the NN (39.58%), CS(45.83%), HZ(47.92%), TH(43.75%), and VN (37.50%) populations and other geographic sources (Supplementary Table S5; Figures 8C,D).

## Discussion

This study profiled the gut microbiota of *C. medinalis* from six geographic sources. More information on the *C. medinalis* gut microbiota could be obtained through MiSeq sequencing than through traditional methods of isolation and culturing (Yang, 2012; Liu et al., 2016). The structure and diversity of the gut microbiota in *C. medinalis* vary across the life cycle of *C. medinalis* (Yang et al., 2020). Recently, we characterized the bacterial structures of *C. medinalis* fed rice or maize for three generations and found that host plant and host plant × generation might have a substantial effect on *C. medinalis* intestinal bacteria based on the PERMANOVA (Yang et al., 2022). In this study, 22 bacterial phyla, 56 classes, 84 orders, 138 families, 228 genera, and 299 species were generated in *C. medinalis* from different geographic sources, including four sites in China, one in Thailand, and one in Vietnam. In the gut microbiota of *C. medinalis* from six geographic sources, the phyla *Proteobacteria* and *Firmicutes* were also the most common bacterial taxa. Alpha diversity indices suggested that bacterial community richness and diversity could be affected by geographic sources. Our findings in this study add to our understanding of the connection between the *C. medinalis* populations and their intestinal microbiota, and will deepen the cognition of the population characteristics of *C. medinalis* and promote the investigation of the *C. medinalis* gut microbiota.

Insect gut microbiota could be shaped by a range of complex factors such as development stages and host plants. In addition, the geographic source was considered as an important factor in many studies on gut microbiota. We analyzed the bacterial configuration of *C. medinalis* from six geographic sources and found that geographic sources could influence the composition of the *C. medinalis* gut microbiota in this work. *Firmicutes* (57.31–93.08% relative abundance) and *Proteobacteria* (4.94–28.66% relative abundance) were the dominant taxa at the phylum level in the gut microbiota of *C. medinalis* from different geographic sources. ANOSIM and PERMANOVA indicated that geographic sources could significantly affect the *C. medinalis* gut microbiota. Similarly, bacterial communities of *Dendroctonus valens* vary with geographic sites in the United States of America, and the bacterial community of the house fly *Musca domestica* varies with geographical location and environment (Adams et al., 2010; Park et al., 2019). Microbiota variations were observed in a vector mosquito *Culex nigripalpus* from various geographic sources (Duguma et al., 2019). Gut microbial communities in indigenous honey bees (*Apis mellifera jemenitica*) from two distinct ecological locations in Saudi Arabia were investigated using 16S ribosomal RNA gene sequencing and culture-dependent methods, and the investigation showed that the relative abundance of the isolated bacteria in native honey bees was high in Al-Baha, with 15 bacterial species (45.5%) specific, and 12 bacterial species (36.4%) were specific to Riyadh (Khan et al., 2017). The discrepancy in the gut microbial diversity of honey bees may be influenced by ontogenetic stage, age, and geographic source (Hroncova et al., 2015). However, Ramalho et al. (2017) discovered that host phylogeny can affect the total bacterial community of spiny ants, while geographic source had no effect. Similar bacterial communities were observed among different populations of *Tuta absoluta* (Meyrick) (Wang et al., 2022). The gut microbial community structures in the bark beetles *D. valens* and *Dendroctonus mexicanus* are obviously stable across geographical sources, and the ANOSIM test indicated no significant changes among the metabolic pathways of the microbial communities in *D. valens* and *D. mexicanus* across geographical sources (Hernández-García et al., 2018). In fact, the geographic source is a complex factor incorporated with biotic and abiotic factors such as host plant, temperature, humidity, and environmental microbes. Coincidentally, these factors with geographic sources assist the insect population with geographic characteristics. In our study, we fed larvae the same rice variety to rule out the interference of the host plant on the variation in gut microbiota of samples from distinct locations. Hu et al. (2020) found that environmental conditions significantly affect the diversity and composition of the *Triatoma rubrofasciata* gut microbiota, and *Staphylococcus* was more common in the laboratory-reared population, while *Enterococcus* was dominant in the wild population. Ge et al. (2021) suggested that host species and geographic location affected the gut microbial communities of the honeybee by altering the relative contribution of assembly processes of the microbial community. Bascuñán et al. (2018) found that the developmental stage of mosquitoes, followed by geographical source, is a more crucial determinant of gut bacterial structure than mosquito species or adult feeding status.

The gut microbiota always has an essential function in the host insect. Essential nutrient provisioning is the primary function of gut bacteria in *Cryptorhynchus lapathi* (L.), followed by digestion and detoxification (Jing et al., 2020). Functional prediction of the gut microbiota revealed that metabolism (70.22–70.53%) was found to be the most functional category in this study, followed by environmental information processing (15.79–17.21%), cellular process (5.29–6.12%), genetic information processing (3.90–4.06%), human diseases (2.32–2.72%), and organismal systems (0.82–0.94%). Likewise, intestinal bacteria in *C. medinalis* fed different host plants perform metabolism most commonly, followed by environmental information processing, cellular process, and genetic information processing according to functional prediction (Yang et al., 2022). Among the top 20 GO functional pathways, the relative abundances of three pathways in the LS population were significantly higher, and 11 pathways in the LS population were significantly lower than those in the other geographic populations. Variation in the relative abundance of the function of the gut microbiota may affect the population characteristics of insects. PERMANOVA and ANOSIM both showed that there were significant variations among the GO pathways of the gut microbiota of *C. medinalis* from different geographic sources. Global and overview maps, carbohydrate metabolism, membrane transport, amino acid metabolism, signal transduction, cellular community—prokaryotes, energy metabolism, nucleotide metabolism, cofactors and vitamins metabolism, and lipid metabolism were the top10 GO functional pathways in the roles of the gut microbiota in *C. medinalis* from six geographic sources. As members of the *Bacilli* class, *Enterococcus* and unclassified *Enterobacteriaceae* are the dominant taxa at the genus level in the gut microbiota of *C. medinalis* from six geographic sources. Many microbes are present in all developmental stages of *C. medinalis*, and *Enterococcus* and unclassified *Enterobacteriaceae* occupy the majority of the bacterial community in larvae, pupae, and adults (Yang et al., 2020). *Enterococcus* and unclassified *Enterobacteriaceae* may be important in the life processes of *C. medinalis*, especially in metabolism. *Enterococcus* has also been reported to function in protecting insects against pathogens, fixing toxins from plants, increasing insect fitness, and tolerating toxic diets (Shao et al., 2011; Johnston and Rolff, 2015; Vilanova et al., 2016; Shao et al., 2017). Additionally, *Enterobacteriaceae* has been documented to be associated with insect metabolism, insect resistance or susceptibility to pathogens and insecticides, and insect adaptation and reproduction (Shi et al., 2012; Wang et al., 2020; Álvarez-Lagazzi et al., 2021; Pers and Hansen, 2021; Polenogova et al., 2021). Host-specific *Enterococcus* could produce dramatically different interactions, and while *Enterococcus* isolated from *Spodoptera frugiperda* facilitates the utilization of a poor diet substrate by *S. frugiperda*, it was obviously antagonistic to *Spodoptera exigua* under the same conditions (Mason et al., 2022). The variation in the relative abundance of pathways of the gut microbiota may be a potentially critical factor in understanding differences in the population characteristics of *C. medinalis*. As important resources, some microbes, such as *B. thuringiensis*, *B. subtilis*, *M. anisopliae*, and *B. bassiana*, have been used in the management of *C. medinalis* (Kandibane et al., 2010; Kirubakaran et al., 2014; Shakir et al., 2015; Ramasubramanian et al., 2022). Gut microbes could be utilized to manage *C. medinalis* by disrupting the makeup of gut microbes, weakening their natural functions, or combining with other control measures. Furthermore, many experiments should be carried out to estimate the effect of the variation in gut microbiota on the population characteristics and validate the function of these microbes, and then more and more functional microorganisms may be incorporated into the strategy of *C. medinalis* management in the future.

In an effort to thoroughly understand the underlying mechanisms of the geographic patterns of the *C. medinalis* gut microbiota, we assessed the phylogenetic signals of ecological processes that could drive microbial communities to diverge (variable selection and dispersal limitation), converge (homogeneous selection and homogeneous dispersal), and drift (the undominated process) (Stegen et al., 2013, 2015). The results indicated that variable selection possesses a significant role within the NN population, and homogeneous selection possesses a significant role within the TH population. Deterministic processes (variable selection and homogeneous selection) and stochastic processes (homogenizing dispersal and dispersal limitation) accounted for 61.11 and 38.89% of the bacterial community turnover between samples within geographic sources, respectively. Variable selection forcefully influenced paired comparisons between the LS population (77.08%) and other geographic sources, and homogenizing dispersal was the dominant ecological process affecting paired comparisons between the NN (39.58%), CS (45.83%), HZ (47.92%), TH (43.75%), and VN (37.50%) populations and other geographic sources. Within or between geographic sources, variable selection and homogeneous dispersal drive the convergence of *C. medinalis* gut bacterial communities, and variable selection, dispersal limitation, and undominated processes drive *C. medinalis* gut bacterial communities to diverge. Homogeneous dispersal probably demonstrated the transfer of gut bacteria between *C. medinalis* individuals within the same geographic population or exposed to the identical environmental microbial pool. Homogeneous selection likely demonstrated the selection of microbiota under the identical inner intestinal environment or external environment. Geography also influences the relative importance of distinct ecological processes in structuring the gut microbial communities of honeybees (Ge et al., 2021). As an important migratory insect pest, the *C. medinalis* population migrates northward from March every year and then southward from September onward (Chang et al., 1980; Riley et al., 1995; Wang et al., 2017). Southeast Asia is considered as the main source area of the *C. medinalis* populations in China (Yan, 2021). Wang et al. (2017) reported that the Nanning population could migrate to the middle and lower reaches of the Yangtze River, including Changsha and Hangzhou. In addition, different locations may have particular weather, and weather could influence the activity of *C. medinalis* (Khan and Ramamurthy, 2004). The LS population harbored a particular geography in Leshan, which is located in the southwestern Sichuan Basin with particular weather surrounding high mountains in southwestern China. The special geography may constrain the communication and migration of *C. medinalis*, making the sources of the LS population complicated. Our study indicated that the ecological processing of gut microbiota in the LS population differed from those in other populations. The LS population, with a special geographic environment, may cause variations in the ecological processing of the gut microbiota with other populations. Although microbes differ among the developmental stages of *C. medinalis*, some microbes, including *Enterococcus*, can be found across all the *C. medinalis* stages (Yang et al., 2020). A recent study indicated that the host plant also affects the microbiota of *C. medinalis* along with the insect generation (Yang et al., 2022). Communication between populations and geography may be the crucial factor affecting the ecological processing of the microbiota in *C. medinalis*. In total, the bacterial communities within geographic sources of *C. medinalis* were mainly determined by stochastic processes, and the bacterial communities between geographic sources were mainly determined by deterministic processes. In the *Helicoverpa armigera* caterpillars, the structure of the gut microbiota is mainly determined by stochastic processes (Li et al., 2022). Therefore, considering the importance of the microbiota, gut microbial shifts in *C. medinalis* across geography may influence the characteristics of the *C. medinalis* populations. Nevertheless, more experiments should be carried out to clarify the assembly and function of the *C. medinalis* gut microbiota.

## Conclusion

Geography may be quite important to determining the *C. medinalis* gut microbiota. The alpha diversity indices of *C. medinalis* gut microorganisms could be influenced by geographic source. PERMANOVA and ANOSIM indicated that the *C. medinalis* gut bacteria could be significantly affected by geographic sources. The most dominant function of the *C. medinalis* intestinal microbiota is metabolism, followed by environmental information processing, cellular process, genetic information processing, human diseases, and organismal systems. PERMANOVA and ANOSIM both revealed significant differences among the GO pathways of the gut microbiota of *C. medinalis* from different geographic sources. In total, the bacterial communities within geographic sources were mainly determined by stochastic processes, and the bacterial communities between geographic sources were mainly determined by deterministic processes. Furthermore, more research should be carried out to elucidate the role of these microbes, which will facilitate the screening of novel targets for the *C. medinalis* management. The findings in this work contribute to a better knowledge of the microbial diversity associated with migratory pests, enrich the character of *C. medinalis* populations, lay a theoretical groundwork for future research on *C. medinalis* gut microbes, and shed light on the population relationship and the shaping of the gut microbiota in *C. medinalis*.

## Data availability statement

The datasets presented in this study can be found in online repositories. The names of the repository/repositories and accession number(s) can be found at: https://www.ncbi.nlm.nih.gov/, PRJNA838393, SRR19216016-SRR19216021.

## Author contributions

YY, YL, and ZL contributed to conceptualization of the study. YY, JG, and ZL contributed to funding acquisition. XL investigated the study. YY, HX, and ZL contributed to the methodology. YL and ZL contributed to supervision. YY, JG, and XL visualized the study. YY wrote the original draft and contributed to writing, reviewing, and editing the manuscript. All authors have read and agreed to the published version of the manuscript.

## Funding

This research was funded by the earmarked fund for China Agriculture Research System, grant number CARS-01, and Zhejiang Provincial Natural Science Foundation of China, grant numbers LY20C140004 and LQ22C140006.

## Conflict of interest

The authors declare that the research was conducted in the absence of any commercial or financial relationships that could be construed as a potential conflict of interest.

## Publisher’s note

All claims expressed in this article are solely those of the authors and do not necessarily represent those of their affiliated organizations, or those of the publisher, the editors and the reviewers. Any product that may be evaluated in this article, or claim that may be made by its manufacturer, is not guaranteed or endorsed by the publisher.

## Supplementary material

The Supplementary material for this article can be found online at: https://www.frontiersin.org/articles/10.3389/fmicb.2022.1035644/full#supplementary-material

Supplementary Figure S1 Rarefaction curves of the bacterial community of *Cnaphalocrocis medinalis* from different geographic sources based on Illumina MiSeq sequencing data.

## Footnotes

^1^ http://qiime.org/

^2^ http://drive5.com/uparse/

^3^ http://www.drive5.com/uchime/uchime_download.html

^4^ http://rdp.cme.msu.edu/

^5^ https://www.r-project.org/

^6^ https://www.r-project.org/

^7^http://huttenhower.sph.harvard.edu/galaxy/ (accessed on 22 March 2022).

^8^ https://github.com/stegen/Stegen_etal_ISME_2013/blob/master/bNTI_Local_Machine.r

^9^ https://github.com/stegen/Stegen_etal_ISME_2013/blob/master/Raup_Crick_Abundance.r
