# Supplementary figures and images for: Gut bacterial communities and their assembly processing in Cnaphalocrocis medinalis from different geographic sources
Source: Front Microbiol. 2022 Dec 15;13:1035644. doi: 10.3389/fmicb.2022.1035644 (PMC9797858; doi:10.3389/fmicb.2022.1035644)

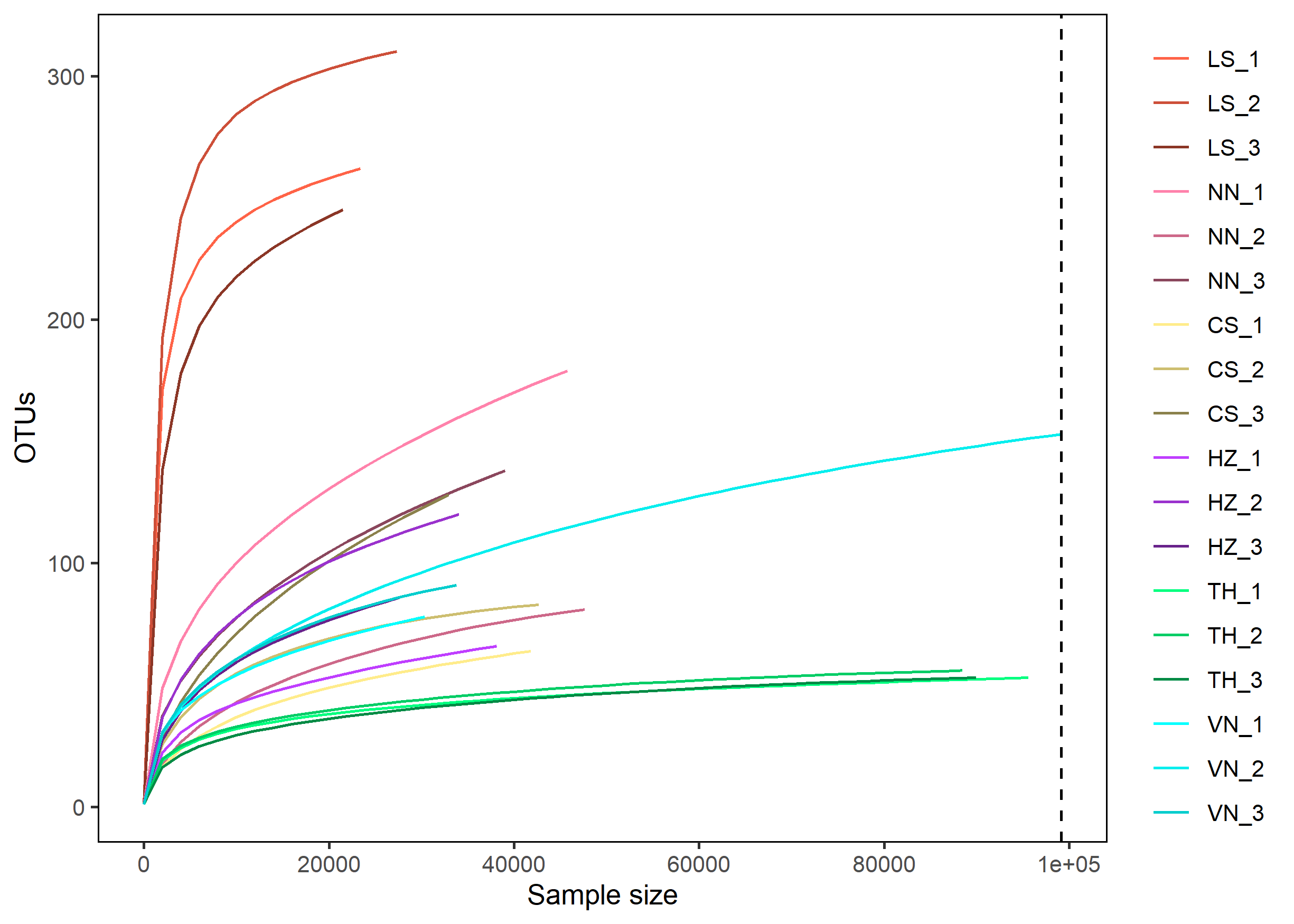

Supplement: Supplementary Figure S1 — Rarefaction curves of the bacterial community of Cnaphalocrocis medinalis from different geographic sources based on Illumina MiSeq sequencing data. [file Image_1.TIFF]
